# *Annulonemertes* (phylum Nemertea): when segments do not count

**DOI:** 10.1098/rsbl.2007.0306

**Published:** 2007-08-07

**Authors:** Per Sundberg, Malin Strand

**Affiliations:** Department of Zoology, Göteborg UniversityPO Box 463, 405 30 Göteborg, Sweden

**Keywords:** phylogenetic analysis, 18S rDNA, Bayesian analysis, nemertean systematics, segmentation

## Abstract

We estimated the phylogenetic position of the pseudosegmented ribbon worm *Annulonemertes minusculus* to test proposed evolutionary hypotheses to explain these body constrictions. The analysis is based on 18S rDNA sequences and shows that the species belongs to an apomorphic clade of hoplonemertean species. The segmentation has no phylogenetic bearing as previously discussed, but is a derived character probably coupled to the species' interstitial habitat.

## 1. Introduction

Berg (1985) described a small interstitial monostiliferous nemertean (phylum Nemertea), *Annulonemertes minusculus*, which he placed in a new genus. One of the most distinguishing characters is the body being externally divided into segments by distinct constrictions. This segmentation is also repeated in the digestive system where the intestine is divided into barrel-shaped segments, which exactly follows the external annulation. Berg (1985) concludes that this is reminiscent of metameric segmentation and asks if the character has any bearing on the phylogenetic position of Nemertea, even though he concludes that since neither the body wall musculature nor the coelom (assuming there are coelomic parts) are segmented, this is a case of a pseudometameric species. Berg argues that the annulation may be either plesiomorphic character to the nemerteans as a clade that could imply a relationship to metameric protostomes, or an apomorphy for this species/genus. Annelids and nemerteans have some sperm structures in common (Whitfield 1972) which could indicate a common ancestry, a view supported by the ‘segmentation’ in *Annulonemertes* if that was to be considered homologous. The interpretation of various structures in nemerteans as coelom homologues (Turbeville 2002) could also be seen as evidence for a closer association. However, the phylogenetic position of phylum Nemertea among the metazoans is enigmatic with many suggestions. Traditionally, it was considered closely related to Platyhelminthes (e.g. Hyman 1951) but most recent analyses (e.g. Zrzavy *et al.* 1998; Giribet *et al.* 2000; Peterson & Eernisse 2001; Zrzavy 2003; Glenner *et al.* 2004) place nemerteans in an apomorphic position closer to annelids, molluscs and entoprocts (plus some other phyla varying between analyses).

Other *Annulonemertes* (supposedly different) species are reported in the literature (Norenburg 1988; Chernyshev & Minichev 2004) but *A. minusculus* is the only named species in the genus. There are other nemertean species described with similar constrictions (notably *Arenonemertes minutus*, Friedrich 1949; *Nemertellina yamaokai*, Kajihara *et al.* 2000), but neither species have these repeated constrictions observed in *Annulonemertes*. The species is referred to as ‘segmented’ in zoological textbooks and thus enigmatic from a phylogenetic point of view (e.g. Brusca & Brusca 2003). Its segmentation has also been discussed in the context of whether the last common ancestor of bilaterian animals was segmented (Balavoine & Adouette 2003). Giribet (2003) discussed the morphological characters supporting the Articulata versus Ecdyozoa metazoan phylogeny hypotheses and refers to *Annulonemertes* as segmented but at the same time conclude that this kind of serial repetition of structures is found in many phyla and do not really count as true segmentation (see also Scmidt-Rasea *et al.* (1998) for a similar distinction). Still, segmentation in this species is discussed in various scenarios of metazoan phylogeny and relationships. For example, Budd & Jensen (2000) use the segmentation to exemplify that complex body plan features are probably more easily lost than gained (under the assumption that segmentation in *Annulonemertes* is a retained plesiomorphy). Moore & Willmer (1997) discuss the possibility that the segmentation suggest an annelid relationship.

Does the metameric segmentation in *Annulonemertes* have any phylogenetic significance at all? We have sequenced a part of the 18S rDNA gene from a specimen of *Annulonemertes* and used this in a nucleotide-based phylogenetic analysis aimed to establish the position of *Annulonemertes* within the Nemertea vis-à-vis suggested metazoan sister taxa. The hypothesis is that if the segmentation is plesiomorphic we would expect *Annulonemertes* to be in a basal position among the nemerteans. Our results, however, show that the metamerism in *Annulonemertes* is an apomorphic character within the phylum and an autapomorphy for the genus, and has no particular phylogenetic bearing when it comes to placing the nemerteans within the Lophotrochozoa. It is probably instead functional, and may be connected to its interstitial life.

## 2. Material and methods

### (a) Specimens and DNA extraction

A specimen of *A. minusculus* was sampled from 8 m depth, sand with some organic material, just by Grötholmen close to the Tjärnö Marine Biological Laboratory, Swedish west coast. After external observations and confirmation of the species, the specimen was placed in 70% ethanol. DNA was extracted using QIAamp DNA Mini Kit for tissue (Qiagen, Inc.) following the protocol supplied by the manufacturer.

### (b) Out-group selection

The phylogenetic position of Nemertea among the metazoans is enigmatic (see §1) and the choice of out-group is not obvious. Based on current information, and proposed affinities of *Annulonemertes*, we included species from Sipuncula, Phoronida, Polychaeta and Vestimentifera in the out-group (table 1).

### (c) Sequencing and phylogenetic analysis

Amplification of the 18S rDNA gene was carried out by PCR using a thermal cycler (MJ Research, Inc., PTC-100 Programmable Thermal Controller) and eukaryotic specific primers (Medlin *et al.* 1988; Turbeville *et al.* 1992; Nygren & Sundberg 2003). The gene was either amplified in one region of approximately 1850 base pairs or in two shorter regions of approximately 1000 base pairs each. PCR was performed with up to 10 μl template in a 50 μl volume with final concentrations of 10 mM Tris–HCl, 50 mM KCl, 2 mM MgCl_2_, 0.3 μM of each primer, 100 μM of each dNTP, 2 units (0.04 U μl^−1^) of Taq DNA polymerase (Sigma product no. D6677). Thermal cycling was initiated with 1–2 min of denaturation at 94–95°C followed by 35–60 cycles of 30 s at 94°C, 1 min at 44–50°C and 2 min at 72°C. After cycling, the reaction was ended with an extension phase at 72°C for 7 min. PCR products were purified with QIAquick PCR Purification Kit (Qiagen, Inc.). Sequencing was carried out either with Cy5-labelled primers on an ALFexpress automated sequencer (Pharmacia) following standard procedures, or on a Beckman Coultier CEQ 2000 (Dye Terminator Cycle Sequencing) following standard procedures with the exception of primer concentration 5 μM instead of the recommended 1.6 μM.

Sequences of the 18S rDNA gene were edited with Lasergene (DNASTAR, Inc.) and aligned in MegAlign using the Clustal W slow-accurate option with pairwise alignment parameters gap/gap length penalties set to 15/6.66 (default values). One thousand seven hundred and thirty base pairs (bp) of the 18S rDNA gene were sequenced for *A. minusculus*. The alignment is available upon request from the corresponding author. Phylogenetic analyses were carried out using Bayesian inference performed with MrBayes v. 3.06 (Huelsenbeck & Ronquist 2001). We used the default values of one cold and three heated Markov chains with invariant sites and gamma distribution (model GTR+I+G, allowing sites to vary independently) according to Modeltest v. 3.6 (Posada & Crandall 1998). Three separate analyses were run to ensure congruence. In each analysis, the Monte Carlo Markov chain (MCMC) length was 1 000 000 generations with sampling of every 100th generation chain. Log-likelihood values for sampled trees stabilized after approximately 100 000 generations, burnin was set to 2000 leaving the last 8000 sampled trees for estimating posterior probabilities (or Bayesian support values).

## 3. Results and discussion

The 18S-based phylogeny (figure 1) places *Annulonemertes* among the hoplonemerteans and in an apomorphic position among the nemerteans. The phylogeny establishes the monophyly of Hoplonemertea and shows that Palaeonemertea is paraphyletic, which corresponds to the general picture in the molecular phylogenies in Sundberg *et al.* (2001) and Thollesson & Norenburg (2002). In the light of this result, many evolutionary steps are needed if we were still to claim that the segmentation is a plesiomorphic character indicating a relationship with other segmented phyla and thus would tell us something about metazoan relationships. It would require that segmentation has been lost in all other nemertean taxa in a more plesiomorphic position, and also that the stylet, the armature of the proboscis, is a plesiomorphic characters lost in hetero- and palaeonemerteans. Although recent findings of an armed palaeonemertean (Kajihara 2006), the ‘stylet’ appears very different from the kind of armature observed among hoplonemerteans. It is clear, when considering all evolutionary steps needed to explain the loss of armature and segmentation among the nemerteans, that the most parsimonious explanation is that the so called segmentation in *Annulonemertes* is an apomorphic character restricted to this particular group of nemerteans. Previous discussions of the position of *Annulonemertes*, and the phylogenetic significance of the segmentation, have also discussed the homology of these constrictions with similar structures in other nemertean taxa. Chernyshev & Minichev (2004) argued that this character was homologous with a similar structure in *N. yamaokai* (Kajihara *et al.* 2000) and assumed that they could be related. Berg (1985) concluded that the only other hoplonemertean species possessing some superficial resemblance to *A. minusculus* is *A. minutus*. However, Berg found so many differences between the two species that he did not hesitate to erect a new genus and species. Unfortunately, we have not been able to include any *Arenonemertes* in the analyses, and we could thus not establish any possible relationships between these two genera.

Our conclusion is that the external constrictions are not phylogenetically informative, but are convergent structures in those hoplonemertean species mentioned earlier. All these species are found interstitially and it may be that the body constrictions are specializations to increase friction against sand grains. Furthermore, the body constrictions are not accompanied with segmentation in inner structures and could not be defined as segmentation according to Scholtz (2002). *Annulonemertes minusculus* may be a somewhat odd looking nemertean, but it does not give any further clues to the placement of nemerteans among the lophotrochozoan protostomes.

## Figures and Tables

**Figure 1:**
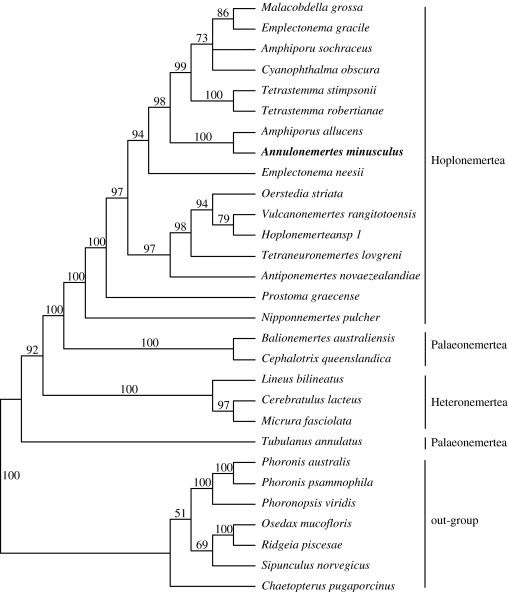
Majority rule consensus tree for the Bayesian analysis using the model GTR+I+G model with sites varying independently. Numbers above branches are posterior probabilities. The species under discussion, *A. minusculus*, is marked in bold.

**Table 1:** List of nemertean species included in the phylogenetic analyses with accession numbers.

species	accession no.
*in-group*	
Heteronemertea	
*Cerebratulus lacteus*, Leidy (1851)	AY145368
*Lineus bilineatus*, Renier (1804)	DQ279932
*Micrura fasciolata*, Ehrenberg (1828)	AY340436
Palaeonemertea	
*Balionemertes australiensis*, Sundberg, Gibson & Olsson (2003)	AY238988
*Cephalotrix queenslandica*, Sundberg, Gibson & Olsson (2003)	AY238989
*Tubulanus annulatus*, Montagu (1804)	AY210452
Hoplonemertea	
*Annulonemertes minusculus*, Berg (1985)	EU011245
*Malacobdella grossa*, Müller (1776)	AY039670
*Tetrastemma robertianae*, McIntosh (1873)	AY928372
*Tetrastemma stimpsonii*, Chernyshev (2003)	AY928376
*Amphiporus allucens*, Bürger (1895)	AY928343
*Amphiporus ochraceus*, Verrill (1873)	AY039668
*Oerstedia striata*, Sundberg (1988)	AY928354
*Prostoma graecense*, Böhmig (1892)	AY039666
*Emplectonema neesii*, Örsted (1843)	AY928348
*Emplectonema gracile*, Johnston (1837)	AY928347
*Cyanophthalma obscura*, Schultze (1851)	AY039667
*Antiponemertes novaezealandiae*, Dendy (1895)	AY928345
*Nipponnemertes pulcher*, Johnston (1837)	AY928352
*Vulcanonemertes rangitotoensis*, Gibson & Strand (2002)	AY928379
*Hoplonemertean* sp. 1^a^	AY928349
*Tetraneuronemertes lovgreni*, Sundberg *et al.* (in press)	AY928350
*out-group*	
Phoronida	
*Phoronis australis*, Haswell (1883)	AF119079
*Phoronis psammophila*, Cori (1889)	AF025946
*Phoronopsis viridis*, Kvitek (1996)	AF123308
Annelida Polychaeta	
*Osedax mucofloris*	AY941263
*Chaetopterus pugaporcinus*	DQ209224
Vestimentifera	
*Ridgeia piscesae*, Jones (1985)	X79877
Sipuncula	
*Sipunculus norvegicus*, Danielssen (1869)	DQ300004

a Pale translucent yellowish-white with 18 dark reddish-brown bands dorsally, several small dark eyes. Approximately 20 mm long. Collected in Hong-Kong.
